# Stable Histone Methylation Changes at Proteoglycan Network Genes Following Ethanol Exposure

**DOI:** 10.3389/fgene.2018.00346

**Published:** 2018-08-30

**Authors:** David P. Gavin, Joel G. Hashimoto, Nathan H. Lazar, Lucia Carbone, John C. Crabbe, Marina Guizzetti

**Affiliations:** ^1^Jesse Brown Veterans Affairs Medical Center, Chicago, IL, United States; ^2^Department of Psychiatry, Center for Alcohol Research in Epigenetics, University of Illinois at Chicago, Chicago, IL, United States; ^3^Department of Behavioral Neuroscience, Oregon Health and Science University, Portland, OR, United States; ^4^VA Portland Health Care System, Portland, OR, United States

**Keywords:** alcohol withdrawal, alcohol dependence, calcium signaling, ChIP-seq, histone methylation, proteoglycans

## Abstract

Alcohol use disorder (AUD) is a chronic mental illness in which patients often achieve protracted periods of abstinence prior to relapse. Epigenetic mechanisms may provide an explanation for the persisting gene expression changes that can be observed even after long periods of abstinence and may contribute to relapse. In this study, we examined two histone modifications, histone 3 lysine 4 tri-methylation (H3K4me3) and histone 3 lysine 27 tri-methylation (H3K27me3), in the prefrontal cortex of Withdrawal Seizure Resistant (WSR) mice 21 days after 72 h of ethanol vapor exposure. These histone modifications were selected because they are associated with active promoters (H3K4me3) and repressed gene expression in a euchromatic environment (H3K27me3). We performed a genome-wide analysis to identify differences in H3K4me3 and H3K27me3 levels in post-ethanol exposure vs. control mice by ChIP-seq. We detected a global reduction in H3K4me3 peaks and increase in H3K27me3 peaks in post-ethanol exposure mice compared to controls, these changes are consistent with persistent reductions in gene expression. Pathway analysis of genes displaying changes in H3K4me3 and H3K27me3 revealed enrichment for genes involved in proteoglycan and calcium signaling pathways, respectively. Microarray analysis of 7,683 genes and qPCR analysis identified eight genes displaying concordant regulation of gene expression and H3K4me3/H3K27me3. We also compared changes in H3K4me3 and/or H3K27me3 from our study with changes in gene expression in response to ethanol from published literature and we found that the expression of 52% of the genes with altered H3K4me3 binding and 40% of genes with H3K27me3 differences are altered by ethanol exposure. The chromatin changes associated with the 21-day post-exposure period suggest that this period is a unique state in the addiction cycle that differs from ethanol intoxication and acute withdrawal. These results provide insights into the enduring effects of ethanol on proteoglycan and calcium signaling genes in the brain.

## Introduction

Alcohol use disorder (AUD) is a chronic condition where sufferers may relapse even after periods of protracted abstinence (Olive, 2010). Gene expression and epigenetic changes following acute and chronic ethanol use and acute withdrawal have begun to be characterized (Gavin et al., 2016; Pandey et al., 2017). However, the mediators of persisting changes to gene expression caused by 72-h ethanol exposure have not been fully explored. The stable nature of some epigenetic marks could provide mechanisms to account for lasting changes in gene expression that mediate relapse to ethanol use following protracted abstinence (Wiren et al., 2006).

In this study, we examine changes in two durable histone modifications, histone 3 lysine 4 and lysine 27 trimethylation (H3K4me3 and H3K27me3). Histones have a half-life of months, and there is evidence that H3K4 and H3K27 methylation are mitotically inherited (Commerford et al., 1982; Chen and Dent, 2014). These two marks serve countervailing roles with H3K4me3 being present at active promoters and H3K27me3 being associated with repression of transcript elongation (Strahl et al., 1999; Schubeler et al., 2004; Guenther et al., 2007). We decided to focus on these histone modifications because they have the stability to encode changes in gene expression over a prolonged period (Robison and Nestler, 2011).

There is a growing literature regarding the association between histone modifications and ethanol. Studies of patients with alcohol dependence have identified ethanol-induced changes to histone modifications in the brain (Zhou et al., 2011; Ponomarev et al., 2012). In the rat amygdala, acute ethanol increases global and prodynorphin and pronociceptin promoter histone acetylation levels (Pandey et al., 2008; D’Addario et al., 2013), and reduces prodynophin and pronociceptin H3K27me3 binding (D’Addario et al., 2013). Moreover, in the hippocampus, ethanol-induced histone H3 acetylation and H3K4me3 have been found to regulate expression of brain derived neurotrophic factor (BDNF) exons (Stragier et al., 2015). Finally, acute ethanol withdrawal leads to decreased histone acetylation in the rat amygdala (Pandey et al., 2008; Sakharkar et al., 2012; Moonat et al., 2013).

A state of alcohol dependence must be inferred in rodents by the emergence of withdrawal signs when alcohol is discontinued. Most laboratory rodents will not voluntarily drink sufficient alcohol to become physically dependent (Crabbe, 2014). Even when exposure to drinking solutions is extended for months, blood alcohol values rarely reach intoxicating levels (Wahlstrom, 1987). A very common procedure used to produce chronic dependence is to expose animals continuously to vaporized alcohol (Goldstein and Pal, 1971; Rogers et al., 1979; Becker, 2014). Following vapor inhalation exposure to intoxicating blood levels, the subsequent withdrawal signs in rodents parallel those in human alcoholics very closely (Friedman, 1980). For example, depending on the vapor concentration, withdrawal from 72 h exposure to ethanol vapor can be lethal in mice (Goldstein, 1972). We have selectively bred Withdrawal Seizure-Prone (WSP) and -Resistant (WSR) mouse lines to display severe or mild withdrawal handling-induced convulsions, respectively, following 72 h continuous vapor exposure (Kosobud and Crabbe, 1986), and have characterized their withdrawal extensively (Metten and Crabbe, 1996). WSP and WSR mice both show many withdrawal signs, but differ on some due to their genetic constitutions. WSP mice show tremors, seizures, enhanced sensitivity to chemical convulsants, anxiety-like behavior and reduced activity, while WSR mice display enhanced backward walking, Straub tail (Kosobud and Crabbe, 1986) and a tendency to engage in relapse drinking (Hashimoto et al., 2011). In the current study, we exposed WSR to 72 h of ethanol vapor and measured the genome-wide distribution of H3K4me3 and H3K27me3 marks in the prefrontal cortex using chromatin immunoprecipitation followed by sequencing (ChIP-seq) 21 days after ethanol exposure. The prefrontal cortex is a critical site of ethanol’s rewarding effects, wherein it mediates approach/avoidant behavior through its communications with multiple brain regions, such as the nucleus accumbens, ventral tegmental area, and amygdala (Chandler et al., 1993; Harper and Matsumoto, 2005).

This study identifies, for the first time, the locations of differential H3K4me3 and H3K27me3 peaks in mice 21 days after ethanol exposure compared to control mice. We find that genes related to proteoglycans are enriched in H3K4me3 peaks and that genes related to calcium signaling are enriched in H3K27me3 binding, suggesting that these gene networks are differentially epigenetically regulated during protracted withdrawal and may have a role in relapse.

## Materials and Methods

### Animal Subjects and Ethanol Intoxication

The Withdrawal Seizure Resistant (WSR-1, -2) mice, derived from heterogeneous HS/Ibg mice by phenotypic selection for resistance to chronic ethanol withdrawal seizures (Crabbe and Kosobud, 1986), were used for this study. We have previously shown altered signaling of genes related to epigenetic regulation following chronic ethanol exposure and increased relapse drinking in these mice (Hashimoto et al., 2011, 2017). WSR-1 was used for the chromatin-immunoprecipitation followed by massively parallel DNA sequencing (ChIP-seq) experiments, and WSR-1 and -2 were used for microarray expression analysis. Mice were maintained with a lights-on and lights-off cycle at 6 a.m. and 6 p.m., respectively, and a room temperature of 22 ± 1°C. Purina Lab Diet chow and water were available *ad libitum* throughout routine husbandry and during experimentation. All animal procedures were in accordance with the US NIH guide for the care and use of laboratory animals and were approved by the Portland Oregon VAMC IACUC.

Four adult WSR-1 male mice per group (control and ethanol) were used for ChIP-seq analyses (H3K4me3 and H3K27me3 ChIP-seq analyses were carried out on the same animals). Four additional adult WSR-1 male mice per group were used in validation ChIP-qPCR experiments; the animals used in ChIP-seq and the animals used in ChIP-qPCR studies were from the same ethanol exposure studies.

Eight adult WSR-1 and -2 male mice per group (control and ethanol) were used for microarray experiments. For each array hybridization, the RNA from two animals from the same selected line and treatment group was pooled; the number of replicates (*n*) per treatment group is therefore 4. Four additional adult WSR-1 and -2 male mice per group were used in qPCR validation experiments; the animals used in microarray studies and the animals used in qPCR studies were from the same ethanol exposure studies.

Exposure to ethanol vapor was carried out as previously described (Wilhelm et al., 2014). Briefly, mice were injected i.p. with 1.5 g/kg ethanol and 68.1 mg/kg pyrazole HCl (to maintain elevated blood ethanol concentrations, BECs, by inhibiting alcohol dehydrogenase) immediately before being placed in the ethanol chambers. Control animals were injected with 68.1 mg/kg pyrazole HCl and placed in chambers identical to the ethanol chambers but with ambient air circulated instead of vaporized ethanol. Each day, mice were briefly removed from the chambers to record weights, administer 68.1 mg/kg pyrazole HCl, and to obtain a blood sample. BECs were monitored daily by sampling 20 μL of blood from the tail vein with ethanol concentrations determined by gas chromatography (Beadles-Bohling and Wiren, 2006). The WSR line and alcohol exposure paradigm used here has been found to induce dependence as documented in several prior publications (Kosobud and Crabbe, 1986; Hashimoto et al., 2011). After 72 h of ethanol exposure, all animals were returned to normal mouse cages for 21 days with daily monitoring of overall health but no further experimental manipulations.

### ChIP-seq Library Generation, Sequencing, and Analysis

Twenty-one days after cessation of ethanol vapor or control exposure, mice were euthanized and the prefrontal cortex was rapidly isolated, snap-frozen in liquid nitrogen, and stored at -80°C as previously described (Hashimoto et al., 2017) until processing. Each prefrontal cortex was homogenized in a 10 mL Dounce homogenizer in RPMI buffer, cross-linked with a final concentration of 1.6% formaldehyde, and fragmented by sonication using a Bioruptor-Pico (Diagenode, Denville, NJ, United States) to achieve fragment sizes ranging from ∼100 to 500 bp. Protein A/G PLUS-Agarose Beads were used to pre-clear the fragmented chromatin samples for 2 h at 4°C with slow end-over-end rotation. Primary antibody [Millipore Anti-Trimethyl-Histone H3 (Lys4), Cat# 07-473; or Millipore Anti-Trimethyl-Histone H3 (Lys27), Cat# 07-449] was added to supernatant after clearing along with fresh agarose beads and incubated overnight at 4°C with gentle end-over-end rotation. The following day, beads were washed with low salt buffer, high salt buffer, lithium chloride buffer, and TE buffer followed by elution from the beads and DNA isolation using the ChIP DNA Clean and Concentrator Kit (Zymo Research, Irvine, CA, United States). DNA was quantitated using the Quant-iT PicoGreen dsDNA Assay Kit (Thermo Fisher Scientific, Waltham, MA, United States) according to the manufacturers protocol. Sequencing libraries were prepared using the NEBNext ChIP-seq Library Prep Master Mix Set and Multiplex Oligos for Illumina Index Primer Set 1 according to the manufacturer’s protocol using 10 ng of ChIP DNA and size selection for 150 bp inserts (New England Biolabs, Ipswich, MA, United States). Short read sequencing assays were performed by the OHSU Massively Parallel Sequencing Shared Resource.

Sequencing results consisting of 75 bp single-ended reads from the prefrontal cortex of 8 WSR-1 mice (four control and four ethanol-treated) and the pooled reads from the input material from all eight samples were obtained. Adapter sequences were removed using trimmomatic (v0.35) (Bolger et al., 2014), exact read copies were removed using fastx collapser (v0.0.13) (Hannon Lab, 2010), and reads were mapped to the GRCm38/mm10 mouse genome assembly with BWA-mem (v0.7.9.a) (Li and Durbin, 2009). After removing reads with mapping scores below 30 we used the R SPP package (Kharchenko et al., 2008) to calculate strand cross-correlation measures and all samples were exceeding or near recommended thresholds set by ENCODE. Two H3K4me3 samples (one control and one ethanol) were removed from further analysis because they contain a large fraction of duplicated reads compared to the other samples.

For the H3K4me3 and H3K27me3 ChIP-seq analysis, we counted the number of mapped reads, the number of unique mapping positions and estimated the library complexity using the non-redundant fraction (Landt et al., 2012). For genomic tracks of read coverage, we extended reads to the fragment length (150 bp) and computed the Pearson correlation coefficient between the mapping profiles of each pair of samples after removing ‘blacklisted’ regions obtained from the ENCODE consortium (Consortium, 2012). To identify peaks above background we used the irreproducibility discovery rate (IDR) analysis detailed by Li et al. (2011) with an IDR cutoff of 0.01. We then performed a differential binding analysis on the H3K4me3 data using the DiffBind package (v3.2) to run EdgeR (Robinson et al., 2010) with an FDR cutoff of 0.1. Finally, we associated all peaks with RefSeq (Pruitt et al., 2005) genes noting the distance to the nearest gene.

Because H3K27me3 ChIP-seq produces broad peaks that cover larger areas of the genome instead of the narrow peaks seen with H3K4me3 marks, we sequenced an additional Illumina lane of 75 base-pair single-ended reads and combined these reads for each sample. Peaks were called using MACS2 (Zhang et al., 2008; Feng et al., 2012) and the IDR analysis was not performed as this method is not applicable to broad peaks. Instead we used stringent overlap criteria requiring that peaks were found to overlap in all four samples of one condition and zero samples of the other condition.

### RNA Isolation and Microarray Processing

Twenty-one days after cessation of ethanol vapor or control exposure, mice were euthanized and the prefrontal cortex was rapidly isolated, snap-frozen in liquid nitrogen, and stored at -80°C as previously described (Hashimoto et al., 2017) until processing. Total RNA was isolated using RNA STAT-60 (Tel Test Inc., Friendswood, TX, United States) with genomic DNA removal using the DNA-Free RNA Kit (Zymo Research) as described previously (Hashimoto et al., 2017) and RNA integrity was determined using a 1% agarose gel stained with SYBR Gold (Thermo Fisher Scientific, Waltham, MA, United States) and quantitated by UV spectroscopy.

Microarrays were purchased from the National Institute on Aging microarray facility which includes 16,897 features corresponding to 7,683 unique GenBank Gene IDs (Nadon et al., 2005). Complex probe was generated by linear synthesis with 33P-dCTP using SuperScript II Reverse Transcriptase (Thermo Fisher, Waltham, MA, United States) as previously described (Nadon et al., 2005). Complex probes were purified using Biospin P-30 columns (Bio-Rad, Hercules, CA, United States) and labeling efficiency determined using a Bioscan QC-4000 XER (Bioscan, Inc., Washington, DC, United States) and hybridized to arrays overnight with gentle mixing. Probe hybridization was measured using a Cyclone Phosphorimager and OptiQuant version 4.0 (Packard Instrument Company, Downers Grove, IL, United States). Spot identification and intensity measurements were carried out on exported data from OptiQuant using Array-Pro Analyzer 4.5 (MediaCybernetics, Rockville, MD, United States).

Spot hybridization intensities were analyzed using R. The microarray data presented here are part of a larger gene expression study to be published at a later date, which includes 62 total array hybridizations including samples derived from the two sexes of multiple mouse lines at 0 h, 8 h, and 21 days after ethanol exposure. In this study, we present the results from eight arrays that corresponded to the ChIP-seq experiments (i.e., WSR male mice prefrontal cortices 21 days after ethanol exposure). Clones with hybridization intensities below background on more than 10% of the arrays were excluded and batch effects were removed using the ‘sva’ package (Leek et al., 2012). Ethanol regulated genes were determined using the empirical Bayes function of the ‘LIMMA’ package (McCarthy and Smyth, 2009; Ritchie et al., 2015).

### Bioinformatic Analysis of ChIP-seq and Gene Expression

Genes showing differential peaks of H3K4me3 or H3K27me3 in ChIP-seq analysis or identified as differentially regulated by post-ethanol were analyzed separately for Gene Ontology (GO) and pathway enrichment using The Database for Annotation, Visualization and Integrated Discovery (DAVID) v6.8 (Huang et al., 2009). The KEGG pathway schematic with ChIP-seq data integrated into a simplified pathway was created in Cytoscape (3.5.1) using the CytoKegg (1.0.1) application (Shannon et al., 2003). Sequencing data was visualized using the Integrated Genomics Viewer (IGV, 2.4.5).

### Confirmation of ChIP-seq and Microarray Results

Peak regions identified in the ChIP-seq analyses were used in the design of primers using the NCBI Primer-BLAST on-line tools (Ye et al., 2012). Quantitative PCR (qPCR) was run on ChIP DNA using the SsoAdvanced Universal SYBR Green Supermix and CFX96 Real-Time System (Bio-Rad, Hercules, CA, United States) and data is presented as percent of input. Gene expression changes were measured by qRT-PCR using the iTaq Universal SYBR Green One-Step Kit (Bio-Rad, Hercules, CA, United States) using RNA from independent animals from the same studies as those used for the microarray experiment described here. Primers were designed using Primer-BLAST (Ye et al., 2012) for *Alk* and were down-loaded from PrimerBank (Wang et al., 2012) for *Wnt5a*, *Camk2a*, *Dgkb*, and *Ezr*. For each set of qPCR and qRT-PCR primers, primer efficiency was between 90 and 110% and resulted in a discreet single peak during melt analysis.

## Results

### ChIP-seq Analysis

**FIGURE 1:**
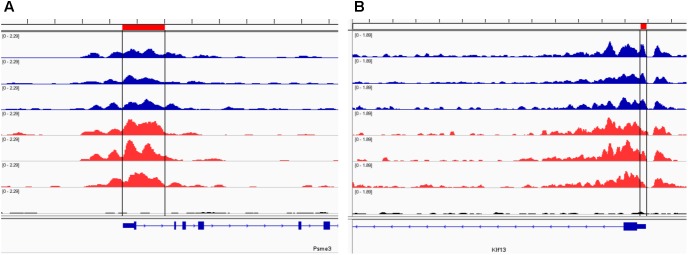
Representative Integrative Genomics Viewer (IGV) browser tracks of H3K4me3 peaks near the TSS following ethanol exposure. For each panel, blue tracks are from control animals, red tracks are from ethanol-treated animals, and black track is the input signal. All IGV tracks in a given comparison have the same scaling factor for the *y*-axis as indicated in the upper left hand region of each track. The region of the genome identified as differentially bound is indicated near the top of each panel in red and with black lines through the tracks. The RefSeq gene map is presented in blue at the bottom of each panel showing the overall gene structure. **(A)** *Psme3* shows increased H3K4me3 in ethanol treated samples. **(B)** *Klf13* shows decreased H3K4me3 in ethanol treated samples at one of the primary peaks at the gene TSS.

Our analysis of the H3K4me3 ChIP-seq data identified 445 differentially bound peaks between the post-ethanol and control mice of which 271 (61%) were located within regulatory regions or gene bodies of 294 unique genes (**Supplementary Table 1**). Seven genes had multiple peaks associated (*1700011I03Rik, Gm10921, Gm13152, Gm14345, Gm14346, Gm38437, Soga1*). Furthermore, 29 peaks had two genes associated, and 4 peaks had three genes associated. We found that 92 (30%) of the gene-associated peaks were located within 100 bp of the transcription start site (TSS; 33 peaks) or proximal promoter region (59 peaks) defined here as 2,000 bp upstream of the TSS and 200 bp into the gene. **Figure 1** shows representative examples of differential Integrative Genomics Viewer (IGV) tracks of H3K4me3 peaks associated with the TSS of two genes in control and post-ethanol mice. Of the genes showing differential H3K4me3 distribution, 52% (159 genes) have been previously shown to be differentially regulated by ethanol (Ponomarev et al., 2012; Wilhelm et al., 2014; Farris et al., 2015; Smith et al., 2016; van der Vaart et al., 2017). The majority of these gene-associated peaks (196, 64%) reported here showed decreased H3K4me3 in the post-ethanol samples compared to control, consistent with prevalent downregulation of gene expression (**Table 1**).

**Table 1:** Matrix of the changes in H3K4me3, H3K27me3, and microarray gene expression and their overlap during ethanol withdrawal in comparison to control.

	H3K27me3/array no change	H3K27me3 peaks present only post-ethanol	H3K27me3 peaks present only in controls	Array increase	Array decrease	Total H3K4me3 changes
H3K4me3/array no change	–	204	36	–	–	
H3K4me3 increase	110	0	0	2		112
H3K4me3 decrease	192	3	0	0	1	196
Array decrease	–	2	0			
Total H3K27me3 changes		209	36			


**FIGURE 2:**
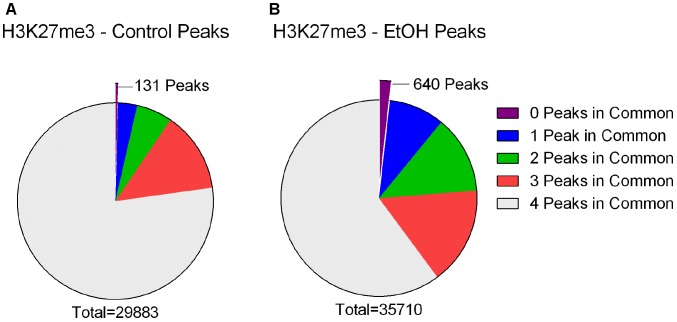
H3K27me3 peaks called in control and ethanol samples. **(A)** Out of a total of 29,883 peaks called in all four Control samples, 23,072 were also present in all four ethanol treated samples (77.21%), 3,972 were present in three of the ethanol treated samples (13.29%), 1,769 were present in two of the ethanol treated samples (5.92%), 939 were present in one of the ethanol treated samples (3.14%), and 131 were present in 0 of the ethanol-treated samples (0.44%). **(B)** Out of a total of 35,710 peaks called in all four ethanol samples, 21,477 were also present in all four Control samples (60.14%), 5,755 were present in three of the control samples (16.12%), 4,570 were present in two of the control samples (12.80%), 3,268 were present in one of the Control samples (9.15%), and 640 were present in 0 of the Control samples (1.79%).

**FIGURE 3:**
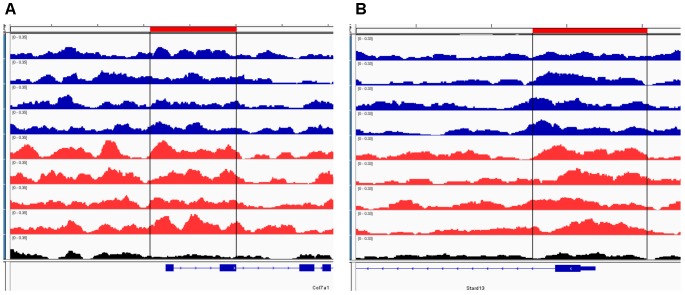
Representative IGV browser tracks of H3K27me3 peaks near the TSS following ethanol exposure. For each panel, the blue tracks are from control animals, red tracks are from ethanol-treated animals, and black track is the input signal. All IGV tracks in a given comparison have the same scaling factor for the *y*-axis as indicated in the upper left hand region of each track. The region of the genome identified as differentially bound is indicated near the top of each panel in red and with black lines through the tracks. The RefSeq gene map is presented in blue at the bottom of each panel showing the overall gene structure. **(A)** *Col7a1* shows increased H3K27me3 in ethanol treated samples. **(B)** *Stard13* shows increased H3K27me3 in ethanol treated samples.

To further our understanding of ethanol dysregulation of histone marks during this 21-day post-ethanol exposure period we also performed ChIP-seq for H3K27me3. H3K27me3 generally produces broad peaks often encompassing several kilobases of DNA, which makes analysis with traditional statistical approaches challenging, requiring a different strategy than the one described above for H3K4me3. We first looked at peaks present in all four samples of each group and found 35,710 peaks in the ethanol treatment group and 29,883 peaks in the control group. A large portion of the peaks identified completely or partially overlapped in the control and ethanol-treatment samples (**Figure 2**). To avoid false-positive results, we used a very stringent method for calling differential H3K27me3 peaks between control and post-ethanol samples. Only when peaks are present in all four replicates of one treatment group with no corresponding peaks present in any of the four replicate samples of the other treatment group do we identify them as differential H3K27me3 peaks. We identified 771 peaks that were present in one condition but not in the other. 640 peaks were present in all four post-ethanol samples but in none of the control samples; 131 peaks were present in all the control samples but in none of the post-ethanol samples (**Figure 2**). Two hundred forty of these differential peaks were associated with 231 unique genes. Five peaks were associated with two genes. Ten genes had two peaks present (*Drd3, Gm5134, Gpr39, Rora, Sorcs2, Svep1, Syt9, Tspan18, Wwox, 2310007B03Rik*), two genes (*Alk, Sncaip*) had three peaks. **Supplementary Table 2** shows the list of the 245 genes associated with H3K27me3 differential peaks (genes with two or three peaks are listed duplicated in the table) and the gene region where the differential peak is located. Eight peaks are associated with the promoter region, eight are in the TSS region, and 229 are associated with the gene body. Of the 245 genes with differentially bound peak regions 85% (209) had H3K27me3 peaks only in the post-ethanol samples compared to control. These results, similarly to the results found for post-ethanol changes on H3K4me3 and described in the previous paragraph, are consistent with prevalent down-regulation of gene expression during the 21-day post-ethanol period (**Table 1**). **Figure 3** shows representative examples of differential IGV tracks of H3K27me3 peaks associated with the TSS of two genes in control and post-ethanol mice. Comparison of our H3K27me3 ChIP-seq data with previously published ethanol data showed 40% (98 genes) of genes identified in this study have been previously identified as regulated by ethanol (Ponomarev et al., 2012; Wilhelm et al., 2014; Farris et al., 2015; Smith et al., 2016; van der Vaart et al., 2017).

Three genes had differential H3K4me3 and H3K27me3 peaks, *Trp63* (transformation related protein 63); *Wnt5a* (wingless-type MMTV integration site family, member 5A); and *Lhfpl3* (lipoma HMGIC fusion partner-like 3). All three genes had reduced H3K4me3 in the ethanol samples and the presence of H3K27me3 peaks in the post-ethanol samples suggesting expression of these genes is repressed during protracted ethanol withdrawal (**Table 1** and **Supplementary Tables 1**, **2**).

### Pathway and Gene Ontology Analyses

**FIGURE 4:**
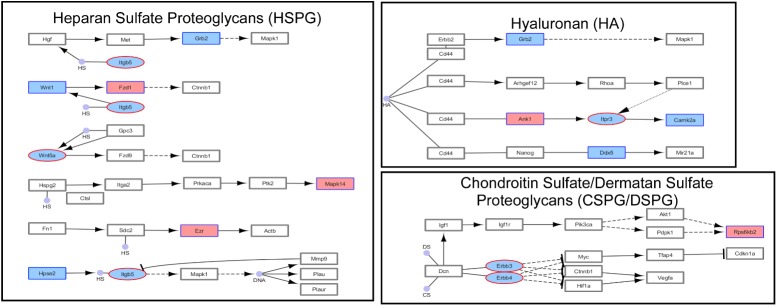
Simplified KEGG pathway “Proteoglycans in Cancer” with H3K4me3 and H3K27me3 enriched genes. The KEGG pathway was down-loaded into Cytoscape (3.5.1) using the CytoKegg (1.0.1) application. ChIP-seq data was overlaid on the pathway with H3K4me3 results shown in blue rectangles and H3K27me3 results shown in red ovals. Blue filled nodes indicate a predicted down-regulation with ethanol based on the ChIP-seq results and pink filled nodes indicate a predicted up-regulation. Proteoglycans play several crucial roles in modulating neuronal migration, axonal regeneration, and synaptic plasticity.

Next we performed pathway and GO analyses on the genes showing differential histone marks. For H3K4me3, the top KEGG pathways identified were “proteoglycans in cancer,” “viral carcinogenesis,” and “viral myocarditis” (**Table 2**). The top GO categories were “dendrite,” “synapse,” and “cell junction” (**Table 3**). Interestingly, four genes that are part of the top KEGG pathway for H3K4me3, “proteoglycans in cancer,” were identified in the H3K27me3 analysis suggesting dysregulation of this pathway through multiple epigenetic mechanisms (**Table 2** and **Figure 4**). For H3K27me3, the top KEGG pathways identified were “calcium signaling pathway” and “nicotinate and nicotinamide metabolism” (**Table 2**). We observed an additional six genes in the “calcium signaling pathway” in our H3K4me3 analysis suggesting an important role of calcium signaling during this post-ethanol period (**Table 2**). The top GO categories for H3K27me3 were “calcium ion binding,” “receptor complex,” and “transcriptional activator activity, RNA polymerase II core promoter proximal region sequence-specific binding” (**Table 3**).

**Table 2:** Enriched KEGG pathways in genes with post-ethanol-induced changes in H3K4me3 and H3K27me3.

KEGG identity	Count	*P*-Value	Genes	Count	*P*-Value	Genes
Proteoglycans in cancer	5	0.0869	Erbb3, Itgb5, Wnt5a, Itpr3	10	0.0009	Ddx5, Hpse2, Wnt5a, Rps6kb2, Camk2a, Grb2, Mapk14, Ezr, Wnt1, Ank1
Calcium signaling pathway	7	0.0100	Htr7, Erbb3, Erbb4, Itpr3, Mylk, Plcd4, Ryr1	6	0.0503	Pde1c, Camk2a, Atp2b2, Cacna1e, Ntsr1, Grin1
Gastric acid secretion	3	0.0542	Slc26a7, Itpr3, Mylk	9	0.0110	Ep300, Jak1, Tradd, Casp8, Ccnd2, Grb2, H2-T23, H2-T9, H2-Bl
Wnt signaling pathway	2	0.4898	Wnt5a, Nfatc1	6	0.0186	Ccnd2, Ctbp1, Wnt5a, Camk2a, Ep300, Wnt1
Phosphatidylinositol signaling system	2	0.3130	Itpr3, Plcd4	4	0.0566	Inpp4b, Dgkb, Pi4k2a, Pik3c2b
Taste transduction	4	0.0157	Scnn1b, Itpr3, Htr3b, Pkd1l3	1	0.7082	Pde1c
Long-term potentiation	1	0.5361	Itpr3	4	0.0162	Gria1, Camk2a, Ep300, Grin1
Prolactin signaling pathway				4	0.0216	Ccnd2, Grb2, Mapk14, Socs7
Thyroid hormone synthesis	3	0.0490	Pax8, Itpr3, Creb5	1	0.8267	Ep300


*Gene names in red indicate decreased gene expression would be predicted based on the histone modification and green indicate increased gene expression would be predicted based on the histone modification.*

**Table 3:** Enriched GO categories in genes with post-ethanol-induced changes in H3K4me3 and H3K27me3.

GO term	H3K27me2		H3K4me3	
	Count	*P*-Value	Count	*P*-Value
Membrane (GO:0016020)	–	–	124	0.0000
Positive regulation of transcription from RNA polymerase II promoter (GO:0045944)	22	0.0010	12	0.3556
Cell junction (GO:0030054)	–	–	27	0.0000
Calcium ion binding (GO:0005509)	22	0.0000	4	0.0000
Dendrite (GO:0030425)	–	–	24	0.0000
Synapse (GO:0045202)	–	–	23	0.0000
Chromatin binding (GO:0003682)	–	–	20	0.0000
Transcriptional activator, RNA polymerase II core promoter proximal region sequence-specific binding (GO:0001077)	11	0.0004	3	0.3832
Receptor complex (GO:0043235)	10	0.0000	2	0.5332
Neuronal cell body (GO:0043025)	2	0.3173	7	0.0006
Z disk (GO:0030018)	7	0.0015	1	0.6353


### Gene Expression Analysis

Additionally, we carried out gene expression array analysis of the PFC of control and post-ethanol exposure mice. We found that 21 d after ethanol exposure 86 of the 7,683 genes present in the microarray platform were differentially expressed, with an unadjusted *p*-value < 0.01, 40% of the differentially expressed genes were downregulated and 60% were upregulated (**Table 3**). Notably, DNA methyltransferase 3A (*Dnmt3a*) was down-regulated during protracted withdrawal from alcohol and has been previously identified as dysregulated following ethanol exposure (Smith et al., 2016; van der Vaart et al., 2017). We then subjected the differentially expressed genes to pathway and GO analyses. The top KEGG pathway identified was “cell adhesion molecules.” The top GO categories were “protein binding,” “nucleoplasm,” and “coronary vasculature development.” The microarray platform used for this experiment contained 49% of the genes that showed changes in H3K4me3 and 18% of the genes that showed changes in H3K27me3. Five genes displaying changes in H3K4me3 or H3K27me3 also showed differential expression; in all five cases, the expression profile of the genes matched the predicted expression based on histone methylation with increased H3K4me3 associated with increased expression and increased H3K27me3 associated with decreased expression. Specifically, we found that genes *Pard3* and *Plagl1* showed increased H3K27me3 in ethanol samples and decreased expression compared to controls; the gene *Calu* displayed reduced H3K4me3 binding and reduced gene expression; genes *Ezr* and *Dgkb* had increased H3K4me3 and increased gene expression (**Tables 1**, **4** and **Supplementary Tables 1**, **2**).

**Table 4:** Post-ethanol-induced gene expression differences in the PFC of male WSR mice.

Symbol	Gene ID	Log FC	*p*-Value	Symbol	Gene ID	Log FC	*p*-Value
**Pard3**	93742	–0.302	0.0084	Lmbrd2	320506	0.154	0.0098
Acbd5	74159	–0.298	0.0078	Erc2	238988	0.157	0.0060
Col4a5	12830	–0.288	0.0009	Psph	100678	0.159	0.0006
Cbln1	12404	–0.273	0.0045	Rbbp8	225182	0.160	0.0016
Smg5	229512	–0.242	0.0061	Ndst2	17423	0.164	0.0072
Pcif1	228866	–0.223	0.0003	Ccno	218630	0.166	0.0071
Mrps18b	66973	–0.215	1.4E-05	Ccpg1	72278	0.168	0.0088
Cul1	26965	–0.197	0.0074	Cdc7	12545	0.171	0.0027
Folr4	64931	–0.194	0.0093	Vps29	56433	0.172	0.0056
Fgf22	67112	–0.192	0.0032	Sri	109552	0.172	0.0027
Dnmt3a	13435	–0.190	0.0019	Emilin1	100952	0.173	0.0018
Jam2	67374	–0.189	0.0021	Gna14	14675	0.174	0.0087
Tnfaip1	21927	–0.189	0.0050	Chd4	107932	0.178	0.0099
Srrt	83701	–0.187	0.0084	Def8	23854	0.179	0.0092
Scp2	20280	–0.184	0.0098	Lars	107045	0.180	0.0030
**Plagl1**	22634	–0.180	0.0067	Fbxo8	50753	0.182	0.0089
Lyst	17101	–0.179	0.0003	Icosl	50723	0.182	0.0051
Ccdc122	108811	–0.174	0.0020	Arl1	104303	0.187	0.0026
Adam19	11492	–0.173	0.0023	Prpsap1	67763	0.193	0.0025
**Calu**	12321	–0.166	0.0053	Med13	327987	0.194	0.0040
Pnpla7	241274	–0.164	0.0072	Usp16	74112	0.198	0.0066
Jam3	83964	–0.155	0.0066	Lasp1	16796	0.201	0.0017
Lsr	54135	–0.153	0.0045	Il17rc	171095	0.204	0.0003
Rps15	20054	–0.152	0.0016	Fam195b	192173	0.205	0.0031
Smarca4	20586	–0.152	0.0021	Kif7	16576	0.205	0.0007
Cd34	12490	–0.151	0.0025	**Ezr**	22350	0.208	0.0003
Efna5	13640	–0.147	0.0039	Reep5	13476	0.210	0.0002
Kif16b	16558	–0.143	0.0035	Tsen15	66637	0.210	0.0049
Trmt2a	15547	–0.135	0.0035	Usp9x	22284	0.213	0.0096
Lgals1	16852	–0.135	0.0024	Hat1	107435	0.218	0.0040
Casp8ap2	26885	–0.133	0.0055	Clk2	12748	0.223	0.0003
Bach2	12014	–0.131	0.0093	Lrp1	16971	0.227	0.0014
Mettl16	67493	–0.129	0.0052	Sf3a2	20222	0.233	0.0031
Hspd1	15510	–0.119	0.0059	Rnaseh1	19819	0.233	0.0072
Ripply3	170765	0.108	0.0069	Zfp229	381067	0.236	0.0002
Psmd2	21762	0.110	0.0088	Ildr1	106347	0.242	0.0004
Olfr976	258364	0.136	0.0086	Alas1	11655	0.245	0.0008
Irak1	16179	0.142	0.0099	Ntrk2	18212	0.259	0.0014
Wbp7	75410	0.147	0.0059	Osbp	76303	0.265	0.0009
Bok	51800	0.147	0.0035	Uck2	80914	0.269	0.0044
Phip	83946	0.149	0.0037	Ndufb7	66916	0.270	0.0035
Eef1b2	55949	0.151	0.0039	**Dgkb**	217480	0.325	0.0017
Heatr5b	320473	0.153	0.0032	Exosc5	27998	0.459	0.0012


*Genes with altered expression after 72-h vapor ethanol exposure followed by 21 days without ethanol in the PFC of male WSR mice. Genes in bold were also identified in ChIP-seq analysis as enriched in either control or ethanol treated samples.*

### Validation Experiments

We validated some of the changes observed in H3K4me3 and/or H3K27me3 analysis using ChIP followed by qPCR analysis of immunoprecipitated DNA in four additional samples per condition. Furthermore, we used qRT-PCR to validate differential expression detected by microarray and measure RNA expression of two genes (*Alk* and *Camk2a*) not present in the microarray platform but displaying H3K4me3 and/or H3K27me3 changes by ChIP-seq.

The five genes selected for validation were: *Alk*, *Wnt5a, Camk2a, Ezr, and Dgkb. Alk* was selected for validation as several previous studies have implicated this gene in modulating alcohol-drinking behavior (Heberlein et al., 2010; Lasek et al., 2011; Dutton et al., 2017); *Wnt5a*, *Camk2a*, and *Ezr* were selected for validation because components of the “Proteoglycans in Cancer” pathway identified as significantly altered by ethanol withdrawal by KEGG pathway analysis of our ChIP-seq results (**Figure 4**). In addition, *Wnt5* displayed a concordant increase in H3K27me3 and decrease in H3K4me3 while *Ezr* and *Dgkb* displayed a concordant increase in H3K4me3 and in gene expression (**Table 4**).

**FIGURE 5:**
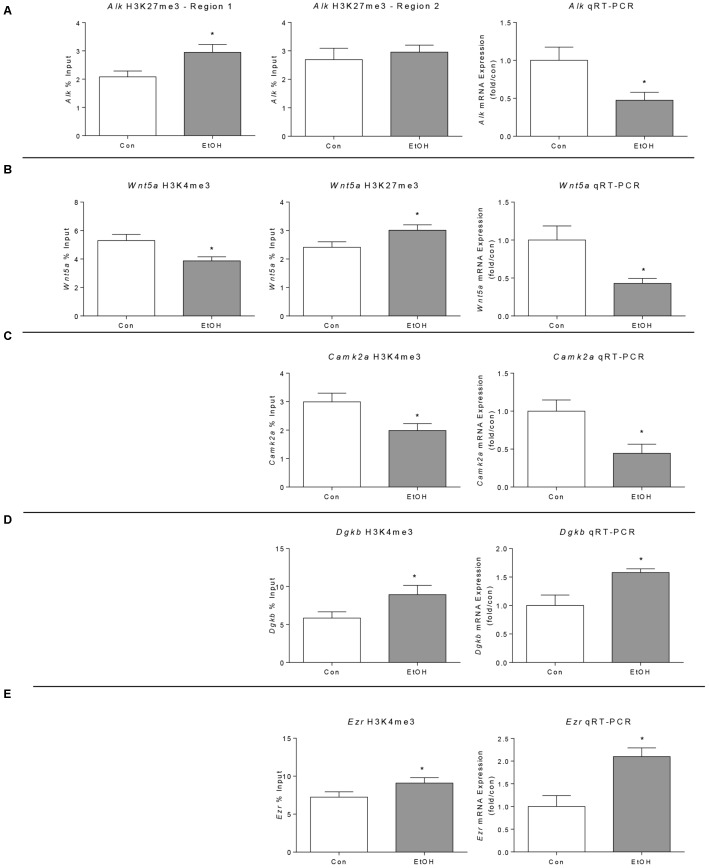
Validation of selected ChIP-seq and microarray results in the prefrontal cortex of WSR male rats 21 days after ethanol exposure by ChIP and qPCR analyses. **(A)** *Alk* genomic region 1 was significantly enriched for H3K27me3 in ethanol samples but region 2 was not significantly enriched. *Alk* gene expression was down-regulated by ethanol exposure as predicted by the H3K27me3 ChIP-seq analysis (*Alk* was not present in the microarray platform used in this study). **(B)** *Wnt5a* H3K4me3 was significantly decreased and H3K27me3 was significantly increased by ChIP-qPCR, in agreement with the ChIP-seq findings. In addition, expression analysis showed decreased *Wnt5a* expression. **(C)** *Camk2a* H3K4me3 was reduced by alcohol confirming ChIP-seq results; in agreement with this modification, its expression was also reduced (*Camk2a* was not present in the microarray platform used in this study). **(D)** *Dgkb* H3K4me3 and gene expression were increased in alcohol withdrawal samples analyzed by ChIP-qPCR and qPCR respectively, confirming the ChIP-seq and microarray results. **(E)** *Ezr* H3K4me3 and gene expression were increased in alcohol withdrawal samples analyzed by ChIP-qPCR and qPCR respectively, confirming the ChIP-seq and microarray results. ^∗^*p* < 0.05 by Student’s *t*-test.

We performed ChIP-qPCR for two regions of the *Alk* gene showing H3K27me3 differential peaks (region 1 at 538 kb downstream from the TSS and region 2 located 520 kb upstream of the TSS) but only found a significant difference for one of the regions (**Figure 5A**, left and middle). Consistent with the presence of the repressive mark, qRT-PCR revealed that *Alk* expression is reduced by ethanol withdrawal (**Figure 5A**, right). The ChIP-qPCR results of *Wnt5a* confirmed the ChIP-seq results, displaying increased H3K27me3 and decreased H3K4me3 (**Figure 5B**, left and middle) and its expression was decreased by ethanol, in agreement with its chromatin state (**Figure 5B**, right). In agreement with the ChIP-seq results, we observed a decrease in *Camk2a* H3K4me3, associated with a decrease in *Camk2a* gene expression measured by qRT-PCR (**Figure 5C**). Finally, we validated the increase in H3K4me3 in the *Ezr* and *Dgkb* genes associated with increased expression (**Figures 5D,E**), in agreement with ChIP-seq and microarray results.

## Discussion

The current study examined stable changes in H3K4me3 and H3K27me3 following protracted withdrawal from ethanol. We performed ChIP-seq experiments in the prefrontal cortex of animals that 21 days previously had been exposed to ethanol. We found 445 H3K4me3 peaks and 771 H3K27me3 peaks that differed between control and ethanol treated mice, indicating persistent changes in chromatin state 21 days following high dose ethanol treatment. In particular, we observed that 64% of the H3K4me3 peaks that differed between the treatment group and the controls were reduced while 85% of the H3K27me3 peaks were increased, indicating a global repression of gene expression. Three genes showed simultaneous reduction of H3K4me3 and appearance of H3K27me3 in the ethanol-withdrawn samples: *Trp63*, *Wnt5a*, and *Lhfpl3*. We also found that five genes that were differentially expressed in our microarray analysis also displayed changes in H3K4me3 or H3K27me3 (**Tables 1**, **4**). All changes in expression were in the direction that would be predicted based on the histone modifications.

We also compared changes in H3K4me3 and H3K27me3 to prior studies that examined gene expression and, interestingly, found that 52% (159 genes) of our differentially tri-methylated H3K4 and 40% (98 genes) of our differentially tri-methylated H3K27 genes have been previously identified as regulated by ethanol (Ponomarev et al., 2012; Wilhelm et al., 2014; Farris et al., 2015; Smith et al., 2016; van der Vaart et al., 2017). For example, we find H3K27me3 peaks following treatment in Glial cell line-derived neurotrophic factor (*Gdnf*) and Anaplastic lymphoma kinase (*Alk*) genes, and increased H3K4me3 peaks in the ethanol samples at the Glutamate Receptor, Ionotropic, AMPA 1 (*Gria1*) gene. These genes have been reported to be dysregulated by ethanol exposure (Carnicella et al., 2009; Heberlein et al., 2010; Lasek et al., 2011; Wolen et al., 2012; Reynolds et al., 2015; Dutton et al., 2017). With regard to H3K4me3 analysis, there is 0.004% chance of observing this level of overlap with the literature findings by chance using a hypergeometric test, suggesting a high level of correlation between H3K4me3 and gene expression. Notably, this overlap is despite myriad different models and ethanol exposure paradigms used in these prior studies. For example, the Ponomarev et al. (2012) and Farris et al. (2015) studies were conducted using human post-mortem samples, while Smith et al. (2016) used mice exposed to chronic intermittent ethanol exposure and measured gene expression 0 h, 8 h, 72 h, and 7 days post-treatment. On the other hand, we found that there is a high chance of randomly observing the level of overlap seen in the H3K27me3 analysis (58%).

We found that “proteoglycans in cancer” and “calcium signaling pathways” emerged as networks affected by ethanol during protracted withdrawal in the ChIP-seq analysis of H3K4me3 and H3K27me3, respectively. Ethanol and its metabolites, such as acetaldehyde have been shown to interfere with the synthesis, stability, and degradation of glycoconjugates, including proteoglycans in the brain and periphery (Waszkiewicz et al., 2012). Proteoglycans serve essential roles in the brain as part of the extracellular matrix (ECM). Most of the cells in the brain secrete ECM proteins, which provide structural support but can also activate or inhibit cell signaling involved in neuronal plasticity. The ECM can be divided into three main compartments: the basement membrane, the neural interstitial matrix, and perineuronal nets (PNs) (Lau et al., 2013; Lasek, 2016). The basement membrane is a component of the blood brain barrier, and the interstitial matrix and PNs help stabilize neural circuits and diffusion rates of membrane receptors, neurotransmitters, and ions (Celio et al., 1998; Yamaguchi, 2000; Pizzorusso et al., 2002; Gogolla et al., 2009; Dityatev et al., 2010; Gundelfinger et al., 2010; McRae and Porter, 2012). Several recent studies have indicated an important role for ECM factors in alcoholism (Zhang et al., 2014; Lasek, 2016).

Prior to the current study the status of ECM components and regulatory factors during protracted ethanol withdrawal were not well characterized. In the current study, we found *Wnt5a*, *Wnt1*, and *Grb2*, and integrin protein *Itgb5* had histone modifications consistent with reduced gene expression. WNT signaling pathway is of particular interest considering the important role it plays in neuritogenesis (Ille and Sommer, 2005). In relation to the ECM, WNT1 has been shown to positively regulate a key component of PNs, aggrecan, in human adipose stem cell culture (Wang and Fawcett, 2012; Luo et al., 2013). In accord with an increase in H3K27me3 at *Wnt5a* reported here, ethanol treatment of neural stem cells reduced *Wnt5a* expression (Vangipuram and Lyman, 2012; Mandal et al., 2015a). WNT5A has been shown to induce the expression of the γ2 subunit of laminin, an important ECM protein in the brain parenchyma and in the epithelial basement membrane (Yamamoto et al., 2009). On the other hand, WNT1 and WNT5A promote the expression of enzymes that degrade the ECM, such as matrix metalloproteinases (MMPs) (Wu et al., 2007; Huang et al., 2017). The epidermal growth factor receptor adapter protein GRB2 also induces MMP expression (Crampton et al., 2009). In addition, we found an increase in H3K4me3 at the *Mapk14* gene. MAPK14 promotes interstitial matrix component fibronectin mRNA expression in hepatic stem cells (Gu et al., 2016). Prior studies indicated that acute or chronic ethanol, for the most part, promote ECM degradation (Guizzetti et al., 2010; Giordano et al., 2011), in part perhaps due to increases in MMP-9 activity (Wright et al., 2003). A reduction of *Wnt* and *Grb2* expression, together with increased *Mapk14*, as indicated by histone modifications found here, would be consistent with the notion that protracted ethanol withdrawal constitutes a period of ECM repair and growth.

Calcium signaling emerged as an overrepresented pathway in our pathway analyses of H3K4me3 and H3K27me3. Blocking certain types of calcium channel signaling has been shown to reduce rodent ethanol consumption (De Beun et al., 1996; Walter and Messing, 1999). In an ethanol exposure paradigm similar to the one used in this study in which rats were exposed to intermittent ethanol and their prefrontal cortex was analyzed 21 days after the last exposure, ion channels, including those that influence neurotransmitter release through effects on membrane potentials and calcium flux, emerged as one of the top GO categories in a miRNA and a DNA methylation study (Tapocik et al., 2013; Barbier et al., 2015). Calcium signaling also emerged as an important pathway following maternal binge-like ethanol doses in embryonic day 18 fetuses in a microarray study (Mandal et al., 2015b). Our results therefore further indicate an important role for calcium signaling in AUD, and suggest epigenetic mechanisms may encode persisting alterations in this pathway.

Some prior studies have examined H3K4me3 and H3K27me3 in relation to ethanol intake. Similar to our results, these studies have indicated that protracted withdrawal from ethanol produces global reductions in H3K4me3 levels (Govorko et al., 2012; Bekdash et al., 2013), although in these studies exposure was done *in utero* and measurements were done in the adult brain. In neonatal mice ethanol was found to increase H3K27me2 levels between 4 and 24 h following treatment (Subbanna et al., 2013). In contrast to our results, one study found that early post-natal ethanol exposure increased H3K4me3 and decreased H3K27me3 at adulthood (Chater-Diehl et al., 2016), and another reported a global increase in H3K4me3 6 h following a single ethanol exposure in adult mice (Finegersh and Homanics, 2014). Additionally, H3K4me3 levels were increased in postmortem human brain samples of subjects with alcoholism (Ponomarev et al., 2012). In light of these previous studies, our results suggest that protracted withdrawal is a unique state differing from controls, ethanol intoxication, and acute withdrawal, in which H3K4me3 remains low and H3K27me3 is high following high-dose ethanol exposure. Our results help clarify the dynamic changes in histone methylation in adulthood caused by ethanol as a function of time after exposure.

We found that differences in H3K4me3 and H3K27me3 in many cases did not lead to differences in gene expression. However, all changes in expression that did occur were in the direction that would be predicted based on the type(s) of chromatin modifications. For instance, we found two genes (*Pard3* and *Plagl1*) with both an H3K27me3 peak in the ethanol-exposed mice and a decrease in expression based on microarray analysis. Moreover, one gene (*Calu*) showed a reduction in H3K4me3 and a decrease in mRNA expression, and two genes (*Ezr* and *Dgkb*) showed an increase in H3K4me3 and mRNA expression. In addition, we decided to validate H3K27me3 and H3K4me3 peaks identified by ChIP-seq in *Alk* and *Camk2a* genes, respectively, and because these two genes were not included in the microarray, we carried out qRT-PCR analysis and found a decreased expression of both genes, as expected by the increased H3K27me3 in *Alk* and the decreased H3K4me3 in *Camk2a*. One reason for the lack of overlap was a methodological limitation of our study as 51% of the genes in which there were H3K4me3 differences and 82% of the genes in which there were H3K27me3 differences were not present in the microarray platform used. A second reason may be inherent to the nature of the relationship between histone modifications and gene expression. In fact, a lack on concordance between H3K4me3 or H3K27me3 and gene expression has been reported by others (Zhou et al., 2011; Chater-Diehl et al., 2016). Specifically, Henikoff and Shilatifard (2011) argued that histone modifications reflect rather than drive transcriptional activity. This explanation is supported by observations that global loss of H3K4me3 does not cause an overall decline in gene expression. In addition, H3K4me3 is not required for transcription in *in vitro* models. Also, there are many instances in which H3K4me3 levels peak after gene expression has already begun (Pavri et al., 2006; Borde et al., 2009; Clouaire et al., 2012; Kuang et al., 2014). Based on these observations there is evidence that the function of some histone marks is to stabilize gene expression over the long term. Certain histone modifications may mark genes for faster or slower induction of gene expression upon re-exposure to a stimulus or at genes that require frequent or infrequent reactivation (Muramoto et al., 2010; Ding et al., 2012; Mosesson et al., 2014). Therefore, the lack of overlap between histone modifications and gene expression reported here is in fact an interesting observation in itself and its further investigation may contribute to the understanding of the role of histone modifications in gene expression.

## Conclusion

Ours is the first study to examine enduring epigenetic changes following protracted withdrawal in adult mice. These results indicate that even in the fully mature brain prior heavy ethanol exposure can produce enduring changes in chromatin structure. Our results indicate an overall reduction in H3K4me3 and increase in H3K27me3. Several of the genes affected by these changes have been previously implicated in AUD. Finally, our results strongly point to alterations in proteoglycans and calcium channel signaling as persisting changes following ethanol exposure. Roles for ECM-related factors, such as proteoglycans, and calcium signaling in AUD have recently gained prominence due to several recent reports.

## Data Availability Statement

The datasets generated for this study can be found in the Sequence Read Archive (SRP156323) and the Gene Expression Omnibus (GSE117925).

## Author Contributions

MG and JH designed the study. DG, JH, NL, LC, JC, and MG analyzed the data and/or interpreted the results. JC developed the mouse strain and provided the expertise related to ethanol administration. JH prepared ChIP samples. NL and LC performed the initial sequencing data analysis. DG and JH performed the downstream pathway analyses. DG, JH, and MG wrote the manuscript. All authors reviewed the manuscript.

## Conflict of Interest Statement

The authors declare that the research was conducted in the absence of any commercial or financial relationships that could be construed as a potential conflict of interest.
