# Supplementary material for: Stable Histone Methylation Changes at Proteoglycan Network Genes Following Ethanol Exposure
Source: Front Genet. 2018 Aug 30;9:346. doi: 10.3389/fgene.2018.00346 (PMC6125400; doi:10.3389/fgene.2018.00346)
Supplement: Supplementary file 1 [file Table_1.DOCX]

**Supplemental Table 1. Ethanol abstinence-induced H3K4me3 differences in the PFC of male WSR mice**

| Symbol | GID | Fold | p.value | FDR | Peak Region |
| --- | --- | --- | --- | --- | --- |
| **Arhgap25** | 232201 | -3.62 | 2.46E-06 | 0.0029 | Gene |
| **Poldip2** | 67811 | -3.38 | 1.96E-05 | 0.0101 | Promoter |
| **Tmem199** | 195040 | -3.38 | 1.96E-05 | 0.0101 | Gene |
| Lhfpl3 | 269629 | -3.22 | 3.57E-05 | 0.0144 | Gene |
| **Jak1** | 16451 | -3.20 | 2.09E-06 | 0.0028 | Gene |
| **Cmss1** | 66497 | -3.19 | 7.66E-04 | 0.0765 | Gene |
| **Filip1l** | 78749 | -3.19 | 7.66E-04 | 0.0765 | Gene |
| **Spin1** | 20729 | -3.13 | 8.12E-05 | 0.0246 | Gene |
| **Homer1** | 26556 | -3.11 | 7.64E-07 | 0.0014 | Gene |
| **Stard10** | 56018 | -3.09 | 4.97E-07 | 0.0012 | Promoter |
| **Gxylt2** | 232313 | -3.07 | 1.62E-04 | 0.0359 | Gene |
| **Etl4** | 208618 | -3.07 | 7.39E-04 | 0.0761 | Gene |
| **Acoxl** | 74121 | -3.06 | 9.50E-06 | 0.0076 | Gene |
| Arhgap31 | 12549 | -3.03 | 1.67E-05 | 0.0094 | Gene |
| Brinp1 | 56710 | -3.03 | 1.67E-05 | 0.0094 | Gene |
| Slc5a7 | 63993 | -3.01 | 1.46E-04 | 0.0345 | Gene |
| Olfr207 | 257973 | -3.00 | 5.11E-05 | 0.0185 | Promoter |
| Cacna1e | 12290 | -2.99 | 4.43E-04 | 0.0594 | Gene |
| **Tnpo3** | 320938 | -2.97 | 2.29E-04 | 0.0439 | Gene |
| **Cry2** | 12953 | -2.96 | 1.38E-04 | 0.0330 | Gene |
| D930015M05Rik | 320507 | -2.96 | 1.38E-04 | 0.0330 | Gene |
| **Ate1** | 11907 | -2.93 | 3.95E-04 | 0.0579 | Gene |
| Ntsr1 | 18216 | -2.88 | 3.97E-05 | 0.0154 | Gene |
| **Ccdc88a** | 108686 | -2.86 | 1.14E-04 | 0.0300 | Gene |
| **Klhl1** | 93688 | -2.86 | 1.30E-04 | 0.0323 | Gene |
| Shank2 | 210274 | -2.85 | 3.99E-04 | 0.0579 | Gene |
| Eml6 | 237711 | -2.85 | 3.32E-05 | 0.0137 | Gene |
| **Grb2** | 14784 | -2.85 | 4.93E-04 | 0.0609 | Gene |
| Kcnq1 | 16535 | -2.84 | 7.55E-05 | 0.0238 | Gene |
| **Zfyve9** | 230597 | -2.84 | 6.68E-04 | 0.0717 | Gene |
| Anapc7 | 56317 | -2.84 | 1.04E-03 | 0.0870 | Gene |
| 1700011I03Rik | 75444 | -2.82 | 7.34E-04 | 0.0761 | Gene |
| **Ipcef1** | 320495 | -2.81 | 1.40E-03 | 0.0999 | Gene |
| Oprm1 | 18390 | -2.81 | 1.40E-03 | 0.0999 | Gene |
| Pakap | 677884 | -2.81 | 2.47E-06 | 0.0029 | Gene |
| **Palm2** | 242481 | -2.81 | 2.47E-06 | 0.0029 | Gene |
| **Jarid2** | 16468 | -2.79 | 4.15E-04 | 0.0594 | Gene |
| **Mast1** | 56527 | -2.79 | 5.20E-07 | 0.0012 | Gene |
| Casp8 | 12370 | -2.78 | 3.53E-04 | 0.0558 | Gene |
| **Klf12** | 16597 | -2.77 | 7.24E-04 | 0.0759 | Gene |
| 6530403H02Rik | 320739 | -2.76 | 6.52E-04 | 0.0704 | Gene |
| **Atp6v1h** | 108664 | -2.76 | 8.94E-05 | 0.0259 | Gene |
| **Pde1c** | 18575 | -2.74 | 1.03E-04 | 0.0284 | Gene |
| Robo2 | 268902 | -2.73 | 8.28E-05 | 0.0246 | Gene |
| Basp1 | 70350 | -2.73 | 8.08E-04 | 0.0769 | Gene |
| Gga2 | 74105 | -2.73 | 3.64E-04 | 0.0563 | Promoter |
| **Zdhhc3** | 69035 | -2.72 | 9.14E-05 | 0.0260 | Gene |
| Syndig1 | 433485 | -2.71 | 1.12E-03 | 0.0897 | Gene |
| Trmt61a | 328162 | -2.68 | 4.35E-04 | 0.0594 | Gene |
| Mkrn2 | 67027 | -2.68 | 1.33E-03 | 0.0982 | Gene |
| Gm21119 | 100861668 | -2.66 | 1.29E-04 | 0.0323 | Gene |
| Asxl3 | 211961 | -2.64 | 5.48E-04 | 0.0647 | Gene |
| Abca4 | 11304 | -2.63 | 1.68E-05 | 0.0094 | Gene |
| **Epha5** | 13839 | -2.62 | 9.32E-04 | 0.0820 | Gene |
| Lsamp | 268890 | -2.60 | 2.63E-04 | 0.0465 | Gene |
| L3mbtl4 | 320858 | -2.59 | 3.64E-04 | 0.0563 | Gene |
| **Trpm2** | 28240 | -2.55 | 9.25E-04 | 0.0820 | Gene |
| **Ccdc171** | 320226 | -2.53 | 3.21E-05 | 0.0134 | Gene |
| **Cd244** | 18106 | -2.53 | 4.43E-04 | 0.0594 | Gene |
| Parp14 | 547253 | -2.52 | 4.41E-04 | 0.0594 | Gene |
| 1700011I03Rik | 75444 | -2.51 | 1.10E-03 | 0.0891 | Gene |
| Pycr1 | 209027 | -2.50 | 3.99E-04 | 0.0579 | Gene |
| Cntnap5a | 636808 | -2.48 | 7.78E-04 | 0.0765 | Gene |
| Satb1 | 20230 | -2.47 | 1.22E-04 | 0.0319 | Gene |
| **Dock8** | 76088 | -2.45 | 1.86E-04 | 0.0396 | Gene |
| Gm38437 | 102465114 | -2.45 | 1.86E-04 | 0.0396 | Gene |
| Opcml | 330908 | -2.45 | 1.22E-03 | 0.0933 | Gene |
| **Zeb2** | 24136 | -2.41 | 1.37E-03 | 0.0990 | Gene |
| Emilin3 | 280635 | -2.39 | 1.38E-03 | 0.0990 | Gene |
| **Gm13152** | 195531 | -2.39 | 1.38E-03 | 0.0990 | Gene |
| **Rab11fip4** | 268451 | -2.39 | 1.38E-03 | 0.0990 | Promoter |
| **Calu** | 12321 | -2.38 | 3.38E-06 | 0.0037 | Gene |
| Gm20754 | 626082 | -2.38 | 7.56E-04 | 0.0765 | Gene |
| 1700011I03Rik | 75444 | -2.37 | 8.61E-04 | 0.0791 | Gene |
| **Hivep3** | 16656 | -2.34 | 3.93E-04 | 0.0579 | Gene |
| **Ubr5** | 70790 | -2.34 | 1.16E-03 | 0.0904 | Gene |
| AV051173 | 100502959 | -2.34 | 2.46E-04 | 0.0451 | Gene |
| **Prdx1** | 18477 | -2.34 | 2.46E-04 | 0.0451 | Promoter |
| Slc9b1 | 74446 | -2.33 | 1.14E-03 | 0.0897 | Gene |
| Tbx20 | 57246 | -2.31 | 1.83E-04 | 0.0394 | Gene |
| Slc35f3 | 210027 | -2.29 | 9.28E-04 | 0.0820 | Gene |
| **Brd3** | 67382 | -2.28 | 6.26E-05 | 0.0210 | Gene |
| **Themis** | 210757 | -2.25 | 8.92E-04 | 0.0807 | Gene |
| **Tdrp** | 72148 | -2.25 | 8.41E-05 | 0.0246 | Gene |
| **Cenpk** | 60411 | -2.23 | 2.41E-05 | 0.0115 | TSS |
| Ppwd1 | 238831 | -2.23 | 2.41E-05 | 0.0115 | Promoter |
| **Trp63** | 22061 | -2.20 | 2.94E-04 | 0.0501 | Gene |
| **Zfp706** | 68036 | -2.20 | 5.38E-05 | 0.0193 | Gene |
| Hnf4g | 30942 | -2.19 | 1.29E-03 | 0.0964 | Gene |
| **Ppp1r37** | 232947 | -2.18 | 1.13E-05 | 0.0080 | Gene |
| Gm38437 | 102465114 | -2.17 | 8.04E-04 | 0.0769 | Gene |
| **Hpse2** | 545291 | -2.17 | 8.04E-04 | 0.0769 | Gene |
| **Myt1l** | 17933 | -2.16 | 2.07E-04 | 0.0423 | Gene |
| Sorbs2 | 234214 | -2.15 | 1.21E-03 | 0.0933 | Gene |
| **Hnrnpf** | 98758 | -2.10 | 9.30E-06 | 0.0076 | Gene |
| **Cap1** | 12331 | -2.09 | 8.29E-04 | 0.0777 | Gene |
| **Gm13152** | 195531 | -2.09 | 1.28E-03 | 0.0960 | Gene |
| **Trps1** | 83925 | -2.09 | 1.30E-03 | 0.0967 | Gene |
| **Urb2** | 382038 | -2.09 | 7.65E-04 | 0.0765 | Gene |
| **Lpcat1** | 210992 | -2.07 | 9.67E-04 | 0.0829 | Gene |
| **Trim46** | 360213 | -2.06 | 9.93E-05 | 0.0275 | Gene |
| **Ajap1** | 230959 | -2.05 | 1.18E-03 | 0.0918 | Gene |
| **Incenp** | 16319 | -2.04 | 7.73E-07 | 0.0014 | Promoter |
| Gm9871 | 207157 | -2.03 | 6.71E-04 | 0.0717 | Gene |
| Olfr846 | 258279 | -2.02 | 1.20E-03 | 0.0925 | Promoter |
| **Susd4** | 96935 | -2.01 | 1.30E-03 | 0.0967 | Gene |
| **Skor1** | 207667 | -1.99 | 9.06E-05 | 0.0260 | Gene |
| Ccdc184 | 239650 | -1.97 | 5.71E-06 | 0.0055 | Gene |
| **Psmc2** | 19181 | -1.95 | 5.47E-04 | 0.0647 | Gene |
| Dennd1a | 227801 | -1.93 | 9.70E-04 | 0.0829 | Gene |
| **Otud6b** | 72201 | -1.90 | 3.99E-04 | 0.0579 | Gene |
| **Dpy19l3** | 233115 | -1.90 | 8.61E-04 | 0.0791 | Gene |
| Gm38437 | 102465114 | -1.90 | 7.40E-04 | 0.0761 | Gene |
| Ep300 | 328572 | -1.86 | 4.45E-04 | 0.0594 | Gene |
| Dlk2 | 106565 | -1.84 | 1.00E-05 | 0.0076 | Gene |
| **Slc25a36** | 192287 | -1.83 | 4.25E-04 | 0.0594 | Gene |
| **Eif4g2** | 13690 | -1.82 | 4.56E-04 | 0.0594 | Promoter |
| Gm20755 | 626410 | -1.82 | 1.13E-03 | 0.0897 | Gene |
| **Spen** | 56381 | -1.82 | 7.74E-04 | 0.0765 | Gene |
| Brca1 | 12189 | -1.81 | 8.89E-04 | 0.0807 | Gene |
| **Fam155a** | 270028 | -1.81 | 1.67E-05 | 0.0094 | Gene |
| **Camk2a** | 12322 | -1.79 | 1.07E-03 | 0.0886 | Gene |
| Wnt5a | 22418 | -1.78 | 4.40E-05 | 0.0165 | Gene |
| Slc30a9 | 109108 | -1.76 | 1.37E-03 | 0.0990 | Gene |
| Hiat1 | 15247 | -1.76 | 4.63E-04 | 0.0594 | Gene |
| **Fbxo3** | 57443 | -1.76 | 2.74E-05 | 0.0123 | Gene |
| Atf6 | 226641 | -1.75 | 9.11E-04 | 0.0816 | Gene |
| **Stt3b** | 68292 | -1.75 | 5.60E-05 | 0.0196 | Gene |
| **Gm13152** | 195531 | -1.73 | 2.32E-04 | 0.0439 | Gene |
| **Wnt1** | 22408 | -1.70 | 3.03E-04 | 0.0511 | Gene |
| Gm10921 | 668963 | -1.70 | 2.13E-04 | 0.0426 | Gene |
| Gm14345 | 630022 | -1.70 | 2.13E-04 | 0.0426 | Gene |
| Gm14346 | 668958 | -1.70 | 2.13E-04 | 0.0426 | Gene |
| Rpl29 | 19944 | -1.66 | 1.90E-04 | 0.0396 | Gene |
| **Rere** | 68703 | -1.62 | 8.90E-04 | 0.0807 | Gene |
| Ubald1 | 207740 | -1.60 | 7.52E-04 | 0.0765 | Gene |
| Wdr35 | 74682 | -1.60 | 6.49E-04 | 0.0703 | Gene |
| Gm21949 | 100505386 | -1.57 | 3.77E-04 | 0.0570 | Gene |
| Iqcj | 208426 | -1.57 | 3.77E-04 | 0.0570 | Gene |
| Gm38437 | 102465114 | -1.56 | 4.21E-05 | 0.0162 | Gene |
| **Pi4k2a** | 84095 | -1.56 | 4.21E-05 | 0.0162 | Promoter |
| Pdk3 | 236900 | -1.55 | 1.01E-03 | 0.0856 | Promoter |
| **9330179D12Rik** | 77558 | -1.55 | 1.29E-03 | 0.0964 | Promoter |
| **Ccnd2** | 12444 | -1.55 | 1.29E-03 | 0.0964 | Gene |
| **Pisd-ps3** | 66776 | -1.53 | 2.33E-04 | 0.0439 | Gene |
| **Gnl3** | 30877 | -1.53 | 7.05E-04 | 0.0747 | Promoter |
| **Pbrm1** | 66923 | -1.53 | 7.05E-04 | 0.0747 | Promoter |
| Snord19 | 100217423 | -1.53 | 7.05E-04 | 0.0747 | Promoter |
| Ahcyl2 | 74340 | -1.53 | 9.91E-04 | 0.0842 | Gene |
| Islr2 | 320563 | -1.52 | 1.25E-04 | 0.0320 | Promoter |
| Vwc2 | 319922 | -1.49 | 1.85E-05 | 0.0097 | Gene |
| **Hnrnpu** | 51810 | -1.48 | 3.56E-04 | 0.0558 | Gene |
| Gm10921 | 668963 | -1.47 | 2.55E-04 | 0.0457 | Gene |
| Gm14345 | 630022 | -1.47 | 2.55E-04 | 0.0457 | Gene |
| Gm14346 | 668958 | -1.47 | 2.55E-04 | 0.0457 | Gene |
| Zc3h4 | 330474 | -1.46 | 3.82E-04 | 0.0571 | Gene |
| **Oasl1** | 231655 | -1.45 | 9.04E-04 | 0.0816 | Promoter |
| Chst5 | 56773 | -1.45 | 5.88E-04 | 0.0670 | Gene |
| Lrrc9 | 78257 | -1.44 | 5.45E-04 | 0.0647 | TSS |
| Xpot | 73192 | -1.44 | 6.82E-04 | 0.0725 | Promoter |
| Lrrc4c | 241568 | -1.42 | 2.19E-04 | 0.0432 | Gene |
| Zswim8 | 268721 | -1.40 | 7.32E-04 | 0.0761 | Promoter |
| **Bsn** | 12217 | -1.39 | 7.65E-04 | 0.0765 | Gene |
| Fras1 | 231470 | -1.36 | 4.42E-04 | 0.0594 | Gene |
| Ssbp1 | 381760 | -1.35 | 4.64E-04 | 0.0594 | Gene |
| **Clip3** | 76686 | -1.33 | 1.27E-04 | 0.0321 | Gene |
| Gm38437 | 102465114 | -1.32 | 2.90E-05 | 0.0127 | Gene |
| **Ldb1** | 16825 | -1.32 | 2.90E-05 | 0.0127 | Gene |
| Kif13a | 16553 | -1.30 | 7.72E-04 | 0.0765 | Promoter |
| Ngef | 53972 | -1.29 | 5.86E-04 | 0.0670 | Gene |
| **Abi1** | 11308 | -1.28 | 1.12E-04 | 0.0298 | Gene |
| Myeov2 | 66915 | -1.28 | 4.27E-04 | 0.0594 | TSS |
| **Ctdsp2** | 52468 | -1.26 | 6.44E-04 | 0.0701 | Gene |
| **Cops4** | 26891 | -1.26 | 3.40E-04 | 0.0547 | Gene |
| Gm38437 | 102465114 | -1.23 | 7.94E-04 | 0.0769 | Gene |
| Got1 | 14718 | -1.23 | 7.94E-04 | 0.0769 | Gene |
| Ptprn2 | 19276 | -1.21 | 5.90E-04 | 0.0670 | Gene |
| **Irgm1** | 15944 | -1.21 | 1.15E-03 | 0.0900 | Gene |
| Mir1983 | 100316716 | -1.20 | 9.37E-04 | 0.0821 | Promoter |
| **Txnl4a** | 27366 | -1.18 | 7.85E-04 | 0.0765 | Gene |
| **Lingo2** | 242384 | -1.16 | 1.09E-03 | 0.0891 | Gene |
| **Atp6v1e1** | 11973 | -1.16 | 1.23E-03 | 0.0936 | Gene |
| **2810408I11Rik** | 69941 | -1.10 | 6.00E-04 | 0.0676 | Promoter |
| **Ccnyl1** | 227210 | -1.10 | 6.00E-04 | 0.0676 | Gene |
| Smcr8 | 237782 | -1.07 | 1.09E-03 | 0.0890 | Promoter |
| Top3a | 21975 | -1.07 | 1.09E-03 | 0.0890 | Gene |
| Pld6 | 194908 | -1.03 | 1.25E-03 | 0.0946 | TSS |
| **Klf13** | 50794 | -0.98 | 1.28E-03 | 0.0963 | TSS |
| Ttl | 69737 | -0.97 | 9.70E-04 | 0.0829 | Gene |
| **Tmem126b** | 68472 | -0.94 | 5.46E-04 | 0.0647 | Promoter |
| **Mbnl2** | 105559 | -0.93 | 4.43E-04 | 0.0594 | Gene |
| **Chl1** | 12661 | -0.91 | 8.54E-06 | 0.0072 | Gene |
| **Akt1s1** | 67605 | -0.72 | 4.55E-04 | 0.0594 | Gene |
| **Tbc1d17** | 233204 | -0.72 | 4.55E-04 | 0.0594 | Promoter |
| **Cep95** | 320162 | -0.63 | 4.69E-04 | 0.0594 | Promoter |
| **Ddx5** | 13207 | -0.63 | 4.69E-04 | 0.0594 | TSS |
| **Baz1b** | 22385 | 0.37 | 7.13E-04 | 0.0752 | TSS |
| Eif5b | 226982 | 0.43 | 1.05E-03 | 0.0879 | Promoter |
| **Txndc9** | 98258 | 0.43 | 1.05E-03 | 0.0879 | Promoter |
| **Psme3** | 19192 | 0.44 | 2.75E-04 | 0.0485 | TSS |
| **Slc1a4** | 55963 | 0.48 | 9.88E-04 | 0.0842 | TSS |
| Ptpn3 | 545622 | 0.57 | 1.38E-03 | 0.0991 | TSS |
| Ctnnd2 | 18163 | 0.63 | 6.06E-04 | 0.0676 | Gene |
| **Pik3c2b** | 240752 | 0.67 | 4.42E-04 | 0.0594 | TSS |
| **Pom121** | 107939 | 0.72 | 3.49E-04 | 0.0556 | TSS |
| **Elmsan1** | 238317 | 0.75 | 2.09E-04 | 0.0424 | Promoter |
| 1700037H04Rik | 67326 | 0.79 | 1.43E-04 | 0.0340 | TSS |
| **Slc6a11** | 243616 | 0.86 | 1.33E-03 | 0.0979 | TSS |
| **Bcar1** | 12927 | 0.93 | 1.25E-04 | 0.0320 | Gene |
| Cbln3 | 56410 | 0.93 | 4.05E-04 | 0.0584 | Promoter |
| Khnyn | 219094 | 0.93 | 4.05E-04 | 0.0584 | TSS |
| **Clcn3** | 12725 | 0.93 | 9.30E-04 | 0.0820 | TSS |
| **Igfbp5** | 16011 | 0.94 | 1.17E-03 | 0.0907 | Gene |
| Fev | 260298 | 0.95 | 9.47E-04 | 0.0827 | Gene |
| Gm11240 | 667768 | 0.97 | 4.46E-04 | 0.0594 | TSS |
| **Rasef** | 242505 | 0.97 | 4.46E-04 | 0.0594 | TSS |
| Fosl2 | 14284 | 1.02 | 4.35E-04 | 0.0594 | Promoter |
| Mir3470a | 100499518 | 1.03 | 8.84E-04 | 0.0807 | Gene |
| Patz1 | 56218 | 1.03 | 8.84E-04 | 0.0807 | Gene |
| **Rps6kb2** | 58988 | 1.04 | 4.96E-04 | 0.0611 | Gene |
| **Lhx2** | 16870 | 1.05 | 2.35E-04 | 0.0439 | Gene |
| **Ezr** | 22350 | 1.09 | 1.10E-03 | 0.0891 | Gene |
| Rab29 | 226422 | 1.14 | 5.59E-04 | 0.0652 | TSS |
| Rap1gap | 110351 | 1.16 | 9.57E-04 | 0.0827 | Gene |
| Npepl1 | 228961 | 1.18 | 9.63E-04 | 0.0829 | TSS |
| Pea15a | 18611 | 1.20 | 9.36E-04 | 0.0821 | Gene |
| **Mbp** | 17196 | 1.21 | 6.14E-04 | 0.0682 | Gene |
| **1700086L19Rik** | 74284 | 1.22 | 4.93E-04 | 0.0609 | Promoter |
| **Slc38a10** | 72055 | 1.23 | 4.62E-04 | 0.0594 | Promoter |
| **Slc12a7** | 20499 | 1.26 | 6.24E-04 | 0.0685 | Gene |
| **Slmap** | 83997 | 1.26 | 3.83E-04 | 0.0571 | Gene |
| **Ctbp1** | 13016 | 1.28 | 4.67E-04 | 0.0594 | TSS |
| Inpp4b | 234515 | 1.33 | 6.04E-04 | 0.0676 | Promoter |
| **Dst** | 13518 | 1.34 | 2.24E-04 | 0.0439 | Gene |
| Lrfn3 | 233067 | 1.34 | 1.49E-04 | 0.0347 | Gene |
| **Hexdc** | 238023 | 1.35 | 3.29E-04 | 0.0535 | TSS |
| Ogfod3 | 66179 | 1.35 | 3.29E-04 | 0.0535 | TSS |
| **Ebf3** | 13593 | 1.36 | 3.13E-04 | 0.0520 | Gene |
| C77080 | 97130 | 1.39 | 1.25E-03 | 0.0946 | Gene |
| Pkd2l2 | 53871 | 1.40 | 3.01E-05 | 0.0129 | Promoter |
| Hepacam | 72927 | 1.41 | 1.34E-03 | 0.0987 | TSS |
| Bhlha9 | 320522 | 1.43 | 6.07E-05 | 0.0208 | Gene |
| **Ganc** | 76051 | 1.43 | 1.67E-04 | 0.0366 | Promoter |
| **Tmem87a** | 211499 | 1.43 | 1.67E-04 | 0.0366 | Promoter |
| **Mapk14** | 26416 | 1.46 | 3.03E-04 | 0.0511 | Promoter |
| **Slc26a8** | 224661 | 1.46 | 3.03E-04 | 0.0511 | Promoter |
| Fsip1 | 71313 | 1.46 | 1.27E-04 | 0.0321 | TSS |
| Gm38437 | 102465114 | 1.47 | 2.80E-04 | 0.0488 | Gene |
| **Tradd** | 71609 | 1.48 | 1.10E-03 | 0.0893 | Gene |
| **Mospd3** | 68929 | 1.49 | 3.13E-04 | 0.0520 | TSS |
| Aars | 234734 | 1.53 | 9.52E-04 | 0.0827 | Gene |
| **Socs7** | 192157 | 1.56 | 2.67E-05 | 0.0122 | Promoter |
| **Atl2** | 56298 | 1.58 | 6.04E-04 | 0.0676 | Gene |
| **Sepp1** | 20363 | 1.58 | 3.72E-04 | 0.0570 | Gene |
| Elfn1 | 243312 | 1.70 | 1.71E-05 | 0.0094 | Gene |
| Smad6 | 17130 | 1.72 | 1.03E-05 | 0.0076 | Promoter |
| **Aco1** | 11428 | 1.72 | 2.64E-05 | 0.0122 | Promoter |
| **Tceal8** | 66684 | 1.73 | 5.57E-05 | 0.0196 | Promoter |
| Lst1 | 16988 | 1.73 | 2.33E-04 | 0.0439 | Gene |
| Npas1 | 18142 | 1.74 | 2.31E-04 | 0.0439 | Gene |
| Ralgapb | 228850 | 1.78 | 9.78E-05 | 0.0273 | Promoter |
| 1600014C23Rik | 72240 | 1.79 | 5.04E-04 | 0.0615 | TSS |
| A530072M11Rik | 100415915 | 1.79 | 1.06E-03 | 0.0884 | Gene |
| **Phf12** | 268448 | 1.79 | 1.25E-03 | 0.0946 | Promoter |
| **Zfp286** | 192651 | 1.81 | 2.54E-04 | 0.0457 | TSS |
| Phb | 18673 | 1.82 | 7.36E-04 | 0.0761 | Promoter |
| Cdc42se1 | 57912 | 1.82 | 6.18E-04 | 0.0683 | Gene |
| **Otud7a** | 170711 | 1.84 | 8.32E-04 | 0.0777 | Gene |
| **Tnrc6b** | 213988 | 1.85 | 3.82E-05 | 0.0150 | Promoter |
| **Grin1** | 14810 | 1.86 | 5.02E-06 | 0.0052 | Gene |
| Dynll2 | 68097 | 1.86 | 1.58E-04 | 0.0359 | Promoter |
| Trpc7 | 26946 | 1.86 | 3.51E-04 | 0.0557 | TSS |
| Cyp4f13 | 170716 | 1.89 | 1.04E-05 | 0.0076 | Promoter |
| **Sema6c** | 20360 | 1.89 | 4.86E-04 | 0.0607 | Gene |
| **Soga1** | 320706 | 1.92 | 7.82E-04 | 0.0765 | Gene |
| Peg13 | 353342 | 1.92 | 2.06E-05 | 0.0104 | Promoter |
| **Trappc9** | 76510 | 1.92 | 2.06E-05 | 0.0104 | Promoter |
| Alpk1 | 71481 | 1.93 | 9.11E-04 | 0.0816 | Gene |
| Gm14164 | 791417 | 2.03 | 2.15E-04 | 0.0429 | Promoter |
| **Trib3** | 228775 | 2.03 | 2.15E-04 | 0.0429 | Gene |
| March10 | 632687 | 2.07 | 7.24E-05 | 0.0231 | Gene |
| **Asap1** | 13196 | 2.18 | 3.40E-05 | 0.0139 | Promoter |
| **Rasgrp2** | 19395 | 2.23 | 1.16E-05 | 0.0081 | TSS |
| **Atp2b2** | 11941 | 2.24 | 1.44E-07 | 0.0005 | Gene |
| **Pnkd** | 56695 | 2.25 | 7.48E-04 | 0.0765 | Gene |
| Tmbim1 | 69660 | 2.25 | 7.48E-04 | 0.0765 | Gene |
| **Dgkb** | 217480 | 2.26 | 1.07E-03 | 0.0885 | Gene |
| **Telo2** | 71718 | 2.30 | 3.84E-04 | 0.0571 | Gene |
| Plxnc1 | 54712 | 2.32 | 3.69E-05 | 0.0147 | Gene |
| **Arpp21** | 74100 | 2.33 | 1.08E-03 | 0.0890 | Gene |
| Sdk2 | 237979 | 2.33 | 4.46E-04 | 0.0594 | Gene |
| Adgrl1 | 330814 | 2.34 | 1.28E-05 | 0.0083 | Gene |
| **Nfix** | 18032 | 2.37 | 1.36E-04 | 0.0328 | Gene |
| **Casz1** | 69743 | 2.40 | 1.71E-06 | 0.0024 | Gene |
| **Soga1** | 320706 | 2.42 | 7.92E-04 | 0.0769 | Gene |
| 8030423F21Rik | 211001 | 2.42 | 3.78E-04 | 0.0570 | TSS |
| **Sptb** | 20741 | 2.47 | 1.23E-03 | 0.0935 | Gene |
| Tmem91 | 320208 | 2.54 | 1.60E-06 | 0.0024 | TSS |
| Six1 | 20471 | 2.54 | 6.35E-05 | 0.0211 | Gene |
| **Gria1** | 14799 | 2.56 | 1.62E-06 | 0.0024 | Gene |
| Cyp3a41b | 100041375 | 2.59 | 5.80E-04 | 0.0670 | Gene |
| **Palmd** | 114301 | 2.60 | 3.61E-04 | 0.0563 | Gene |
| 4931431C16Rik | 74364 | 2.68 | 9.23E-04 | 0.0820 | Gene |
| **Acox3** | 80911 | 2.68 | 9.23E-04 | 0.0820 | TSS |
| **Ank1** | 11733 | 2.73 | 1.12E-03 | 0.0897 | Gene |
| H2-Bl | 14963 | 3.25 | 1.54E-05 | 0.0094 | Promoter |
| H2-T23 | 15040 | 3.25 | 1.54E-05 | 0.0094 | Promoter |
| H2-T9 | 15051 | 3.25 | 1.54E-05 | 0.0094 | Promoter |

Listed are genes displaying differential H3K4me3 binding after chronic EtOH exposure and extended abstinence. Differential immunoprecipitation was identified by FDR q-values < 0.1. Uncorrected p-values are included for comparison. Peak regions were identified within the gene body (Gene; region of transcription), at the transcription start site (TSS; ± 100 bp of a TSS), and within the promoter region (2,000 bp up-stream and 200 bp down-stream of a TSS). Genes previously shown to be regulated by EtOH are **bold** and genes with EtOH regulation in the same direction as predicted by the H3K4me3 ChIP are **bold underlined**. Peaks overlapping with multiple regions (i.e. TSS and Promoter) are shown with the region closest to TSS (i.e. TSS > Promoter > Gene). Genes that are listed more than once in this table were found to have multiple differential H3K4me3 peaks associated with them.
