# Supplementary material for: Stable Histone Methylation Changes at Proteoglycan Network Genes Following Ethanol Exposure
Source: Front Genet. 2018 Aug 30;9:346. doi: 10.3389/fgene.2018.00346 (PMC6125400; doi:10.3389/fgene.2018.00346)
Supplement: Supplementary file 2 [file Table_2.DOCX]

**Supplemental** **Table 2. Ethanol abstinence-induced H3K27me3 differences in the PFC of male WSR mice**

| Symbol | GID | Group | Peak Region |
| --- | --- | --- | --- |
| **St18** | 240690 | Ethanol | Gene |
| Cnga3 | 12790 | Ethanol | Gene |
| Aox4 | 71872 | Ethanol | Gene |
| **Pard3b** | 72823 | Ethanol | Gene |
| Erbb4 | 13869 | Ethanol | Gene |
| Plcd4 | 18802 | Ethanol | TSS |
| **Usp37** | 319651 | Ethanol | Gene |
| Nhej1 | 75570 | Ethanol | Gene |
| **Sp100** | 20684 | Ethanol | Gene |
| Iqca | 74918 | Ethanol | Gene |
| **Gpr39** | 71111 | Ethanol | Gene |
| Nckap5 | 210356 | Ethanol | Gene |
| Tnni1 | 21952 | Ethanol | TSS |
| Igfn1 | 226438 | Ethanol | Gene |
| Hmcn1 | 545370 | Ethanol | Gene |
| Fam129a | 63913 | Ethanol | Gene |
| Sell | 20343 | Ethanol | Gene |
| **Plagl1** | 22634 | Ethanol | Gene |
| **Enpp3** | 209558 | Ethanol | Gene |
| Mir466j | 100316671 | Ethanol | Promoter |
| Adamts14 | 237360 | Ethanol | Gene |
| Gstt4 | 75886 | Ethanol | Gene |
| Gm5134 | 333669 | Ethanol | Gene |
| Gm5134 | 333669 | Ethanol | Gene |
| **Btbd11** | 74007 | Ethanol | Gene |
| Slc5a8 | 216225 | Ethanol | Gene |
| **Nr1h4** | 20186 | Ethanol | Gene |
| Erbb3 | 13867 | Ethanol | Gene |
| **Meis1** | 17268 | Ethanol | Gene |
| **Dock2** | 94176 | Ethanol | Gene |
| Ebf1 | 13591 | Ethanol | Gene |
| F630206G17Rik | 100009664 | Ethanol | Gene |
| Olfr56 | 18356 | Ethanol | Gene |
| 5133400J02Rik | 71297 | Ethanol | Gene |
| Obscn | 380698 | Ethanol | Gene |
| Hs3st3a1 | 15478 | Ethanol | Gene |
| Shisa6 | 380702 | Ethanol | Gene |
| Odf4 | 252868 | Ethanol | Gene |
| **Rph3al** | 380714 | Ethanol | Gene |
| **Rbfox3** | 52897 | Ethanol | Gene |
| Atp6v1c2 | 68775 | Ethanol | Gene |
| **Npas3** | 27386 | Ethanol | Gene |
| Rad51b | 19363 | Ethanol | Gene |
| Efcab11 | 78767 | Ethanol | Gene |
| Dnah11 | 13411 | Ethanol | Gene |
| Dcdc2a | 195208 | Ethanol | Gene |
| F13a1 | 74145 | Ethanol | Gene |
| Bmp6 | 12161 | Ethanol | Gene |
| A330033J07Rik | 320614 | Ethanol | Gene |
| Susd3 | 66329 | Ethanol | Gene |
| **Fbp1** | 14121 | Ethanol | Gene |
| Zfp457 | 431706 | Ethanol | Gene |
| Thbs4 | 21828 | Ethanol | Gene |
| **1700029F12Rik** | 66479 | Ethanol | Gene |
| **Nid2** | 18074 | Ethanol | Gene |
| **1700112E06Rik** | 76633 | Ethanol | Gene |
| Wnt5a | 22418 | Ethanol | Gene |
| Dnah1 | 110084 | Ethanol | Gene |
| Prrxl1 | 107751 | Ethanol | Gene |
| Otx2os1 | 606497 | Ethanol | Gene |
| **Adam28** | 13522 | Ethanol | Gene |
| **Scel** | 64929 | Ethanol | Gene |
| Gdnf | 14573 | Ethanol | Gene |
| **Sntb1** | 20649 | Ethanol | Gene |
| **Ly6h** | 23934 | Ethanol | Gene |
| Fam83h | 105732 | Ethanol | Gene |
| Cyth4 | 72318 | Ethanol | Gene |
| Triobp | 110253 | Ethanol | Gene |
| D030018L15Rik | 402773 | Ethanol | Gene |
| **Olfr288** | 545140 | Ethanol | Gene |
| **Myh11** | 17880 | Ethanol | Gene |
| A630010A05Rik | 545280 | Ethanol | Gene |
| **Trp63** | 22061 | Ethanol | Gene |
| **Muc4** | 140474 | Ethanol | Gene |
| Itgb5 | 16419 | Ethanol | Gene |
| Mylk | 107589 | Ethanol | Gene |
| Pla1a | 85031 | Ethanol | Gene |
| **Drd3** | 13490 | Ethanol | Gene |
| **Drd3** | 13490 | Ethanol | Gene |
| Gm813 | 328695 | Ethanol | Gene |
| Grik1 | 14805 | Ethanol | Gene |
| **Sh3bgr** | 50795 | Ethanol | Gene |
| Itpr3 | 16440 | Ethanol | Gene |
| H2-L | 14980 | Ethanol | Gene |
| **Ddx39b** | 53817 | Ethanol | Gene |
| **Pgc** | 109820 | Ethanol | TSS |
| A330050F15Rik | 320722 | Ethanol | Gene |
| **Clip4** | 78785 | Ethanol | TSS |
| **Alk** | 11682 | Ethanol | Gene |
| **Alk** | 11682 | Ethanol | Gene |
| **Alk** | 11682 | Ethanol | Gene |
| Vit | 74199 | Ethanol | Gene |
| **Epas1** | 13819 | Ethanol | Gene |
| C330024C12Rik | 399626 | Ethanol | Gene |
| **Myo7b** | 17922 | Ethanol | Gene |
| Epb4.1l4a | 13824 | Ethanol | Gene |
| Mcc | 328949 | Ethanol | Gene |
| **Sncaip** | 67847 | Ethanol | Gene |
| **Sncaip** | 67847 | Ethanol | Gene |
| **Myo5b** | 17919 | Ethanol | Gene |
| **Zbtb7c** | 207259 | Ethanol | Gene |
| **Slc14a2** | 27411 | Ethanol | Gene |
| Nfatc1 | 18018 | Ethanol | Gene |
| Batf2 | 74481 | Ethanol | Gene |
| Htr7 | 15566 | Ethanol | Gene |
| Sh3pxd2a | 14218 | Ethanol | Gene |
| **Il1rn** | 16181 | Ethanol | Promoter |
| Pax8 | 18510 | Ethanol | Gene |
| Ass1 | 11898 | Ethanol | Gene |
| **C230014O12Rik** | 329387 | Ethanol | Gene |
| Gm13490 | 433426 | Ethanol | Gene |
| Tanc1 | 66860 | Ethanol | Gene |
| **Pla2r1** | 18779 | Ethanol | Gene |
| Pde11a | 241489 | Ethanol | Gene |
| **Ttn** | 22138 | Ethanol | Gene |
| **Tspan18** | 241556 | Ethanol | Gene |
| **Angpt4** | 11602 | Ethanol | Gene |
| Bpifa6 | 545477 | Ethanol | Gene |
| Bpifa2 | 19194 | Ethanol | Gene |
| **Raly** | 19383 | Ethanol | Gene |
| a | 50518 | Ethanol | Gene |
| Myh7b | 668940 | Ethanol | Gene |
| Gm826 | 329554 | Ethanol | Gene |
| **Eya2** | 14049 | Ethanol | Gene |
| **Il7** | 16196 | Ethanol | Gene |
| **Mecom** | 14013 | Ethanol | Gene |
| Frem2 | 242022 | Ethanol | Gene |
| **Hao2** | 56185 | Ethanol | Gene |
| Casq2 | 12373 | Ethanol | Gene |
| Sycp1 | 20957 | Ethanol | Gene |
| **Alx3** | 11694 | Ethanol | Gene |
| 1700123O12Rik | 73624 | Ethanol | Gene |
| **Slc26a7** | 208890 | Ethanol | Gene |
| **Svep1** | 64817 | Ethanol | Gene |
| **Rgs3** | 50780 | Ethanol | Gene |
| **Ror1** | 26563 | Ethanol | Gene |
| Cachd1 | 320508 | Ethanol | Gene |
| 4930407G08Rik | 77223 | Ethanol | TSS |
| Zmynd12 | 332934 | Ethanol | Gene |
| Trim63 | 433766 | Ethanol | Gene |
| Iffo2 | 212632 | Ethanol | Gene |
| **Camta1** | 100072 | Ethanol | Gene |
| Megf6 | 230971 | Ethanol | Gene |
| **Lrrc17** | 74511 | Ethanol | TSS |
| Fbxl13 | 320118 | Ethanol | Gene |
| 6030443J06Rik | 320719 | Ethanol | Gene |
| Lhfpl3 | 269629 | Ethanol | Gene |
| **1700001C02Rik** | 75434 | Ethanol | Gene |
| Sorcs2 | 81840 | Ethanol | Gene |
| Sorcs2 | 81840 | Ethanol | Gene |
| Jakmip1 | 76071 | Ethanol | Gene |
| **Evc** | 59056 | Ethanol | Gene |
| **Bst1** | 12182 | Ethanol | Gene |
| **Lnx1** | 16924 | Ethanol | Gene |
| Rassf6 | 73246 | Ethanol | Promoter |
| Crybb1 | 12960 | Ethanol | Gene |
| Crybb2 | 12961 | Ethanol | Gene |
| Cux2 | 13048 | Ethanol | Gene |
| Hpd | 15445 | Ethanol | Promoter |
| Sdk1 | 330222 | Ethanol | Gene |
| Flt1 | 14254 | Ethanol | Gene |
| Stard13 | 243362 | Ethanol | TSS |
| **Asb4** | 65255 | Ethanol | Gene |
| **Slc25a13** | 50799 | Ethanol | Gene |
| Cped1 | 214642 | Ethanol | Gene |
| **Zc3hav1** | 78781 | Ethanol | Gene |
| **Creb5** | 231991 | Ethanol | Gene |
| **Thnsl2** | 232078 | Ethanol | Gene |
| M1ap | 110958 | Ethanol | Gene |
| **Slc6a13** | 14412 | Ethanol | Gene |
| Abcc9 | 20928 | Ethanol | Gene |
| Vmn1r67 | 171263 | Ethanol | Promoter |
| Ryr1 | 20190 | Ethanol | Gene |
| **Nav2** | 78286 | Ethanol | Gene |
| Rgma | 244058 | Ethanol | Gene |
| Gdpd4 | 233537 | Ethanol | Gene |
| Syt9 | 60510 | Ethanol | Gene |
| Syt9 | 60510 | Ethanol | Gene |
| Abca15 | 320631 | Ethanol | Gene |
| **Scnn1b** | 20277 | Ethanol | Gene |
| Tnfrsf26 | 244237 | Ethanol | Gene |
| 9530052E02Rik | 619321 | Ethanol | Gene |
| **F10** | 14058 | Ethanol | Gene |
| Tex15 | 104271 | Ethanol | Promoter |
| Fgl1 | 234199 | Ethanol | Gene |
| **BC030870** | 407795 | Ethanol | Gene |
| **Slc6a2** | 20538 | Ethanol | Gene |
| Ces1e | 13897 | Ethanol | Gene |
| Ces2c | 234671 | Ethanol | Promoter |
| Pkd1l3 | 244646 | Ethanol | Gene |
| **Hydin** | 244653 | Ethanol | Gene |
| Wwox | 80707 | Ethanol | Gene |
| Wwox | 80707 | Ethanol | Gene |
| Hsd17b2 | 15486 | Ethanol | Gene |
| Cdh13 | 12554 | Ethanol | Gene |
| Trim67 | 330863 | Ethanol | Gene |
| **Pard3** | 93742 | Ethanol | Gene |
| BC049352 | 408059 | Ethanol | Gene |
| **Htr3b** | 57014 | Ethanol | Gene |
| **Megf11** | 214058 | Ethanol | Gene |
| **Rora** | 19883 | Ethanol | Gene |
| **Rora** | 19883 | Ethanol | Gene |
| 5730403I07Rik | 70487 | Ethanol | Gene |
| **1300017J02Rik** | 71775 | Ethanol | Gene |
| Tmem108 | 81907 | Ethanol | Gene |
| **Col7a1** | 12836 | Ethanol | TSS |
| **Fbxw27** | 76998 | Ethanol | Gene |
| Rbms3 | 207181 | Ethanol | Gene |
| Acaa1b | 235674 | Ethanol | Gene |
| 2310007B03Rik | 71874 | Control | Gene |
| 2310007B03Rik | 71874 | Control | Gene |
| **Gpr39** | 71111 | Control | Gene |
| **Capn8** | 170725 | Control | Gene |
| Npffr1 | 237362 | Control | Gene |
| Olfr54 | 18354 | Control | Gene |
| Smoc1 | 64075 | Control | Gene |
| Il17rb | 50905 | Control | Gene |
| 4930503F20Rik | 218902 | Control | Promoter |
| **Gpc5** | 103978 | Control | Gene |
| Krt78 | 332131 | Control | Gene |
| Mucl1 | 20771 | Control | Gene |
| Galnt14 | 71685 | Control | Gene |
| **Sncaip** | 67847 | Control | Gene |
| **Sall3** | 20689 | Control | Gene |
| Glis3 | 226075 | Control | Gene |
| **Tll2** | 24087 | Control | Gene |
| **Tspan18** | 241556 | Control | Gene |
| Ccdc73 | 211936 | Control | Gene |
| Sptlc3 | 228677 | Control | Gene |
| Enpep | 13809 | Control | Gene |
| **Svep1** | 64817 | Control | Gene |
| Myo18b | 74376 | Control | Gene |
| B230112J18Rik | 77846 | Control | Gene |
| **Oas3** | 246727 | Control | Gene |
| **Ocm** | 18261 | Control | Gene |
| **Ckm** | 12715 | Control | Gene |
| **Tmc3** | 233424 | Control | Gene |
| **Stk33** | 117229 | Control | Gene |
| **Itgal** | 16408 | Control | Gene |
| Zfp553 | 233887 | Control | Gene |
| Ppapdc1a | 381925 | Control | Gene |
| 5830411N06Rik | 244234 | Control | Gene |
| Cd209b | 69165 | Control | Gene |
| B930025P03Rik | 320014 | Control | Gene |
| Fli1 | 14247 | Control | Gene |

Genes with differential H3K27me3 binding following chronic EtOH exposure and extended abstinence. Differential immunoprecipitation was identified by regions with overlapping peaks in all four control or EtOH samples with no peaks in the same region in the other comparison group. Peak regions were identified within the gene body (Gene; region of transcription), at the transcription start site (TSS; ± 100 bp of a TSS), and within the promoter region (2,000 bp up-stream and 200 bp down-stream of a TSS). Genes previously shown to be regulated by EtOH are bold and genes with EtOH regulation in the same direction as predicted by the H3K27me3 ChIP are bold underlined. Peaks overlapping with multiple regions (i.e. TSS and Promoter) are shown with the region closest to TSS (i.e. TSS > Promoter > Gene). Genes that are listed more than once in this table were found to have multiple differential H3K27me3 peaks associated with them.
